# Late-Onset ADHD: A Diagnostic Mirage or a New Clinical Frontier? A Systematic Review of the Evidence

**DOI:** 10.1192/bjo.2026.11252

**Published:** 2026-06-30

**Authors:** Seyanne Govender

**Affiliations:** University of Essex, Essex, United Kingdom

## Abstract

**Aims:**

Attention-Deficit Hyperactivity Disorder (ADHD) is a neurological developmental disorder that involves a criterion which requires an onset of symptoms before 12 years of age for clinical diagnosis (American Psychiatric Association, 2013).

Be that as it may, longitudinal studies have identified cohorts of adults with symptoms of ADHD but also lack a clear childhood history of symptoms. This phenomenon challenges the core diagnostic criteria described in the DSM–5 and creates notable uncertainty clinically (Moffitt et al., 2015).

This systematic review aims to critically analyse and evaluate the published evidence that both supports and contradicts the validity of adult-onset ADHD being a diagnostic construct, as well as to investigate other possible causalities for this type of clinical presentation.

**Methods:**

Following PRISMA guidelines (Page et al., 2021), a comprehensive search of PubMed, PsycINFO, and Embase was conducted for peer-reviewed studies that have been published from 2000 onwards, exploring late-onset ADHD. Studies were screened by one reviewer. Due to the heterogeneity of the studies, a narrative synthesis was performed usingthe extracted data to arrange findings into arguments based on the various topics that materialised, pertaining to methodology, aetiology, and differential diagnosis.

**Results:**

The synthesis identified a notable conflict in the evaluated literature. Multiple longitudinal studies provided evidence supporting a late-onset ADHD presenting subtype. Nevertheless, this evidence is countered by literature arguing that findings in support of adult-onset ADHD are attributable to limitations in methodology, including both recall bias when patients self-report retrospectively as well as the failure to detect “masked“ childhood symptoms (Faraone et al., 2016). Moreover, a significant amount of literature explores the idea that there may be an overlap of symptoms with conditions that present similarly to ADHD such as complex trauma, emotional dysregulation and misuse of substances.

**Conclusion:**

The evidence analysed and evaluated for a distinct late-onset neurological developmental subtype of ADHD remains inconclusive. These adult presentations outline a complex reality facing the clinicians of which require further meticulous and thorough assessment. This systematic review shows a need for this late-onset ADHD subtype to be further explored and studied with more subjects, as well as investigating possible strategies that could be implemented to mitigate undiagnosed or misdiagnosed patients. Healthcare professionals should make conducting a comprehensive differential diagnosis and recording collateral history a priority, in order to avoid a patient remaining undiagnosed and to distinguish ADHD from similar presenting and/or mimicking conditions.

